# Managing the Combination of Nonalcoholic Fatty Liver Disease and Metabolic Syndrome with Chinese Herbal Extracts in High-Fat-Diet Fed Rats

**DOI:** 10.1155/2013/306738

**Published:** 2013-02-18

**Authors:** Yi Tan, Weiguo Lao, Linda Xiao, Zhenzhong Wang, Wei Xiao, Mohamed A. Kamal, J. Paul Seale, Xianqin Qu

**Affiliations:** ^1^School of Medical & Molecular Biosciences, University of Technology Sydney, P.O. Box 123, Broadway, NSW 2007, Australia; ^2^Kanion Pharmaceuticals, Lianyungang, Jiangsu 22200, China; ^3^Department of Pharmacology, University of Sydney, NSW 2006, Australia

## Abstract

Nonalcoholic fatty liver disease (NAFLD) is the hepatic manifestation of metabolic syndrome (MetS). The aim of the study was to evaluate the effects of Chinese herbal extracts from *Salvia miltiorrhiza* and *Gardenia jasminoides* (SGE) on the combination of NAFLD and MetS induced by a high-fat diet (HFD) in rats. After 6 weeks of HFD feeding, rats (*n* = 10 each group) were treated with saline, rosiglitazone (RSG), and SGE for 4 weeks. HFD rats were obese, hyperinsulinemic, hyperlipidemic and increased hepatic enzymes with the histological images of NAFLD. Treatment with SGE significantly reduced serum triglycerides (TG), nonesterified fatty acids and enhanced insulin sensitivity, and ameliorated the elevated serum hepatic enzymes compared with HFD-saline group. SGE treatment also attenuated hepatic TG by 18.5% (*P* < 0.05). Histological stains showed SGE decreased lipids droplets in hepatocytes (*P* < 0.05) and normalized macrovesicular steatosis in HFD rats. Significant reduction of TNF-**α** and IL6 in adipose tissue was detected in SGE treated rats. The anti-inflammatory action may be, at least in part, the mechanism of SGE on MetS associated with NAFLD. This study discovered that SGE is capable of managing metabolic and histological abnormalities of NAFLD and MetS. SGE may be an optimal treatment for the combination of NAFLD and MetS.

## 1. Introduction

Non-alcoholic fatty liver disease (NAFLD) is a pathologic entity, including a spectrum of liver damage ranging from simple steatosis to non-alcoholic steatohepatitis (NASH), advanced fibrosis, and progression to cirrhosis [1]. Pathophysiology of NAFLD still has not been completely clarified but a large body of clinical and experimental evidence suggests that ectopic fat deposition in the liver plays a fundamental role in the development and progression of NAFLD [2–4]. The increased hepatocellular lipids are correlated to central obesity, insulin resistance, dyslipidaemia, and impaired glucose tolerance, a cluster of metabolic syndrome (MetS) [5, 6]. The prevalence of NAFLD has reached epidemic proportions in recent years, in parallel with the increasing prevalence of obesity and MetS worldwide. NAFLD is currently conceptualised as the hepatic manifestation of MetS, and it is an early warning sign of future risk for type 2 diabetes and cardiovascular disease [5–8]. 

Given the rising coincidence of MetS and NAFLD, development of an effective treatment for obesity-related NAFLD to prevent future disease-related morbidity and mortality is a priority. To date, intervention for MetS and NAFLD remains mainly lifestyle modifications and no pharmacological treatment has been proven to be effective for NAFLD associated with MetS. The pathogenesis of the combination of NAFLD and MetS is multifactorial, including hepatic insulin resistance, increased ectopic fat deposition in the liver and other non-adipose tissues, and adipocytokines-trigged inflammation [9]. Thus, novel therapy for the disease should be capable of managing insulin resistance, lowing hyperlipidaemia and anti-inflammation as well rendering benefits on liver histological outcomes [10]. 

Chinese Herbal Medicine (CHM) has been used in China and other parts of Asian counties for thousands of years. A special feature of Chinese medicine is the use of a formula containing several herbs (like a cocktail) to ameliorate a set of abnormalities related to a disease. Herbal extracts contain multiple naturally occurring compounds that can target different pathological pathways involved in the disease, providing therapeutic effects via a spectrum of actions. In our previous study, we demonstrated that a Chinese herbal formula, containing a high amount of *Salvia miltiorrhiza *Bge extract, effectively reversed metabolic syndrome in a high-fatdiet (HFD) [11]. The dried root of *Salvia miltiorrhiza (S. miltiorrhiza)* is a Chinese herb commonly included in prescriptions to ischemic heart disease, hyperlipidaemia [12, 13], and chronic liver disease [14]. The fruit of *Gardenia jasminoides (G. jasminoides)* is a Chinese herb for cleaning away toxicity in TCM. Recent study showed that the active ingredient of *G. jasminoides* (geniposide) has an alleviating effect on fatty liver in obese diabetic mice [15]. Based on our previous study and other's findings, we combined the extracts from *S. miltiorrhiza* and *G. jasminoides* and evaluated their effects on the coexisting NAFLD and MetS induced by HFD feeding in rats. 

## 2. Materials and Methods

### 2.1. Preparation of Chinese Herbal Extracts

Chinese herbal extracts of *S. miltiorrhiza* and *G. jasminoides* were prepared by Kanion Pharmaceuticals (Lianyungang, Jiangsu, China). In general, each dried herb was authenticated using microscopic examination to ascertain the species' authenticity. Ground herbs were first extracted for 2 h with 65% aqueous ethanol at room temperature followed by 2 h decoction at 120°C. The result was then vacuum-filtered through a filter paper and concentrated in a rotating vacuum evaporator (Yahya Rong Biochemical Instrument, Shanghai, China) at 40°C. The highly concentrated solution was freeze-dried to obtain a solid powder with a yield of 8% and 10% (powder versus raw herb, w/w) for Salvia root and Gardenia fruit, respectively. The quality control for the herbal extracts was performed using an Agilent 1200 series liquid chromatography/mass selective detector equipped with QTOF 6510 mass spectrometer (Agilent Technologies Inc., CA, USA) with botanical markers of tanshinone IIA for *S. miltiorrhiza* and gypenoside for *G. jasminoides* obtained from the Beijing Institute of Materia Medica. The content of tanshinone IIA and gypenoside in 1 g of herbal extract was 120 mg and 30 mg, respectively. The mixture of *S. miltiorrhiza *and* G. jasminoides *extracts, coded as SGE, was used for animal study. 

### 2.2. Animals and Treatment

Male Sprague-Dawley (SD) rats at 6 weeks of age were supplied by the Animal Resources Centre (Perth, Australia). All experimental procedures were approved by the University of Technology Sydney Animal Ethics Committee, following guidelines issued by the National Health and Medical Research Council of Australia. SD rats were maintained on a 12 h light/dark cycle (lights on 0600) under constant temperature (22°C) with *ad libitum* access to standard chow diet or a high-fat diet (HFD, 59% fat, 21% protein, and 20% carbohydrate by energy composition) for 6 weeks to induce MetS and NAFLD. After 6 weeks of HFD feeding, HFD fed rats were randomly divided into three groups (*n* = 10 each group) to receive saline 5 mL/kg of body weight (HF-Con), rosiglitazone (HFD-RSG, 3 mg/kg of body weight), and SGE (HFD-SGE, 2 g/kg of body weight) by daily oral gavage for 4 weeks. HFD feeding continued during the 4-week treatment period. Meanwhile, saline (5 mL/kg of body weight) was administrated to chow fed rats (chow, *n* = 10) as the model control. 

### 2.3. Determination of Metabolic Parameters, Liver Enzymes, and Insulin Sensitivity

At the end of treatment, blood samples were collected from the tail of rats after an overnight fast (12 hours). Fasting serum total cholesterols (TCs), high-density lipoprotein cholesterol (HDL-C), triglycerides (TGs) and nonesterified fatty acids (NEFAs) were analysed using enzymatic colorimetric kits obtained from Roche Diagnostic (Mannheim, Germany) and Wako Pure Chemical Industries (Japan), respectively. Low-density lipoprotein cholesterol (LDL-C) concentrations were calculated by Friedewald's formula: LDL-C (mmol/L) = TC − HDL-C − TG/2.2 [16]. Fasting serum insulin concentration was measured using a RIA kit (Linco Diagnostic Services). Fasting serum glucose, alanine aminotransferase (ALT), and aspartate aminotransferase (AST) were determined by spectrophotometric analysis using commercial kits (Dialab, Vienna, Austria). All experimental assays were carried out according to the manufacturer's instruction. 

Whole-body insulin sensitivity was estimated using the homeostasis model assessment of insulin resistance (HOMA-IR) by using the formula: [fasting serum glucose (mmol) times fasting serum insulin (mU/mL)]/22.5 [17]. Because HOMA is negatively correlated with insulin sensitivity, low HOMA-IR values indicate high insulin sensitivity, whereas high HOMA-IR values indicate insulin resistance.

### 2.4. Tissue Collection and Measurement of Liver Triglycerides

 At the end of the experiment, animals were anesthetized using inhalant aesthetic gas (isoflurane and nitrous oxide) after 12 h of fasting. Liver and visceral fat (epididymal and peri-rental adipose tissues) were quickly excised, washed by ice-cold PBS, then stored at −80°C for subsequent histological and molecular assays.

Approximately 50 mg of liver tissues was homogenized at 4°C in RIPA lysis buffer (Sigma-Aldrich, St. Luis, MO, USA). Lipids from total liver and muscle homogenate were extracted using chloroform/methanol method (2 : 1), evaporated, and dissolved in 1 mL ethanol (Sigma-Aldrich). TG concentration was assayed using kits from *Roche Diagnostic (Mannheim Germany)* following the manufacturers' instructions.

### 2.5. Measurement of Adipokines

Frozen epididymal fats (200 mg) were homogenized in 1 mL lysis buffer containing 150 mM NaCl, 1 mM PMSF, 10% Glycerol, and the complete protease inhibitor cocktail (Roche Diagnostics, Indianapolis, IN, USA). The homogenates were incubated on ice for 30 min and spun at 10,000 g at 4°C for 10 min. The supernatant was collected for analysis of leptin, necrosis factor-*α* (TNF-*α*), interleukin 6 (IL6) in fat tissue using multiplex rat adipocyte Linoplex kits obtained from Linco Research (Millipore, St. Charles, MO) for the simultaneous quantification of leptin, IL-6, and TNF-*α* according to the manufacturer's instruction. Resulting leptin, TNF-*α*, and IL-6 contents were determined using a multiplex reader (BioRad Bio-Plex 200 System; Bio-rad Laboratories, Hercules, CA, USA) and expressed as nM in mg of fat tissue.

### 2.6. Histological Analysis

A small portion of frozen liver tissue was cut and embedded with precooled optimal cutting compound (Torrance, CA, USA) for cryostat sectioning at 6 *μ*m. The sections were mounted on microscope slides then fixed with 10% formaldehyde solution for 48 h. The samples were then stained with Haematoxylin and Eosin (H&E) or Oil Red O (Sigma-Aldrich) to investigate architecture of the liver and hepatic lipid droplets. Stained Oil Red O (ORO) slides were visualized with the Olympus microscope, and images were captured with an Olympus digital camera (DP70, Tokyo, Japan) using Image-Pro6.2 software (Media Cybernetics, Inc., MD, USA). Lipid droplets was quantified at least 5 different high-power fields in a blinded way. Masson's Trichome (MT) stain was used for evaluation of liver fibrosis. For each group, liver samples from 6 to 8 rats were prepared and stained and six fragments from each liver were analysed. All slides were scanned at an absolute magnification of 200x using Image-Pro6.2 software (Media Cybernetics) under a light microscopy (Olympus, BX51 microscope, Tokyo, Japan).

### 2.7. Statistical Analysis

All values are expressed as the means ± SEM. Comparisons across the four groups were done using one-way ANOVA followed by post-hoc analysis of Tukey's test to determine significant differences between the two groups using Prism version 4 (GraphPad Inc., San Diego, CA, USA). *P*-value <0.05 was considered statistically significant. 

## 3. Results

### 3.1. Effects of SGE on Metabolic Profiles and Insulin Sensitivity

HFD feeding significantly increased body weight and visceral fat mass compared with chow fed rats (Table 1). Weight gain in SGE group was significantly lower than that in HFD control rats and RGS treated rats (*P* < 0.01). SGE treatment also markedly reduced visceral fat mass by 46% (*P* < 0.01). The liver weights were not significantly different amongst three groups of HFD rats, though SGE slightly reduced liver weight. After a total of 10 weeks of HFD feeding, SD rats became mildly hyperglycaemic, and SGE treatment reduced fasting serum glucose levels by 8%, but the reduction did not achieve a statistical significance (Table 1). SGE reduced serum TC, TG, and NEFA levels by 23.2% (*P* < 0.05), 51.4% (*P* < 0.01), and 22.3% (*P* < 0.05), respectively, when compared with the HFD control. LDL-C concentrations were also decreased in SGE-treated HFD-rats (*P* < 0.05). SGE also raised HDL-C levels by 11.6% compared to HFD control, but the statistical significance was not achieved. HFD rat was insulin resistant reflected by hyperinsulinaemia as well as significantly increased value of Home-IR (Table 1). SGE treatment produced a similar effect to RGS on insulin resistance, evidenced by reduction of fasting serum insulin levels and improved HOME-IR (Table 1). 

### 3.2. SGE Reduced Hepatic Triglycerides and Ameliorated the Elevated Liver Enzymes of HFD Rats

**Figure 1:**
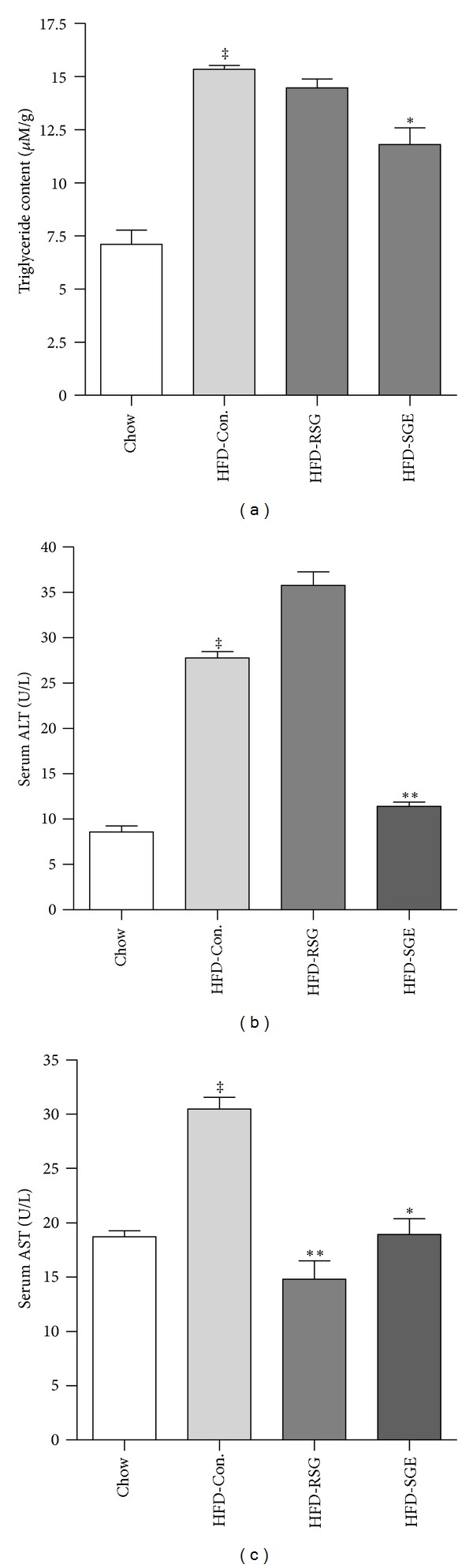
Hepatic triglyceride contents (a), serum alanine aminotransferase (b), and aspartate aminotransferase (c) values in chow fed rats and HFD control rats treated with saline (5 mL/kg of body weight), and HFD rats treated with rosiglitazone (HFD-RSG, 3 mg/kg of body weight) or SGE (HFD-SGE, 2 g/kg of body weight). Data are means ± SEM; *n* = 10 rats/group. ^†^ *P* < 0.05, ^‡^ *P* < 0.01 versus chow control; *P < 0.05 and **P < 0.01 compared with HFD-Con.

Figure 1(a) showed that HFD feeding increased hepatic TG by 2.2-fold compared to chow fed rats (15.34 ± 0.23 *μ*M/g versus 7.11 ± 1.10 *μ*M/g). SGE treatment significantly attenuated the elevated hepatic TG levels by 18.5% (*P* < 0.05, compared with HFD-Con.), and there was no significant reduction of hepatic TG in RSG treated rats (Figure 1(a)). 

Figures 1(b) and 1(c) showed that serum ALT and AST concentrations in HFD fed rats were significantly higher than those in chow fed rats (27.2 ± 1.3 and 31.18 ± 1.66 U/L versus 8.3 ± 0.6 and 16.92 ± 0.79 U/L, resp.). The RSG treatment had further increased ALT levels (36.22 ± 1.30 U/L). In contrast to RSG, SGE treatment significantly attenuated the elevated ALT and AST values to 11.01 ± 0.61 U/L (*P* < 0.01 versus HFD-Con.) and 17.3 ± 2.1 U/L (*P* < 0.05 versus HFD-Con.). 

### 3.3. Effects of SGE on Adipokines

**Figure 2:**
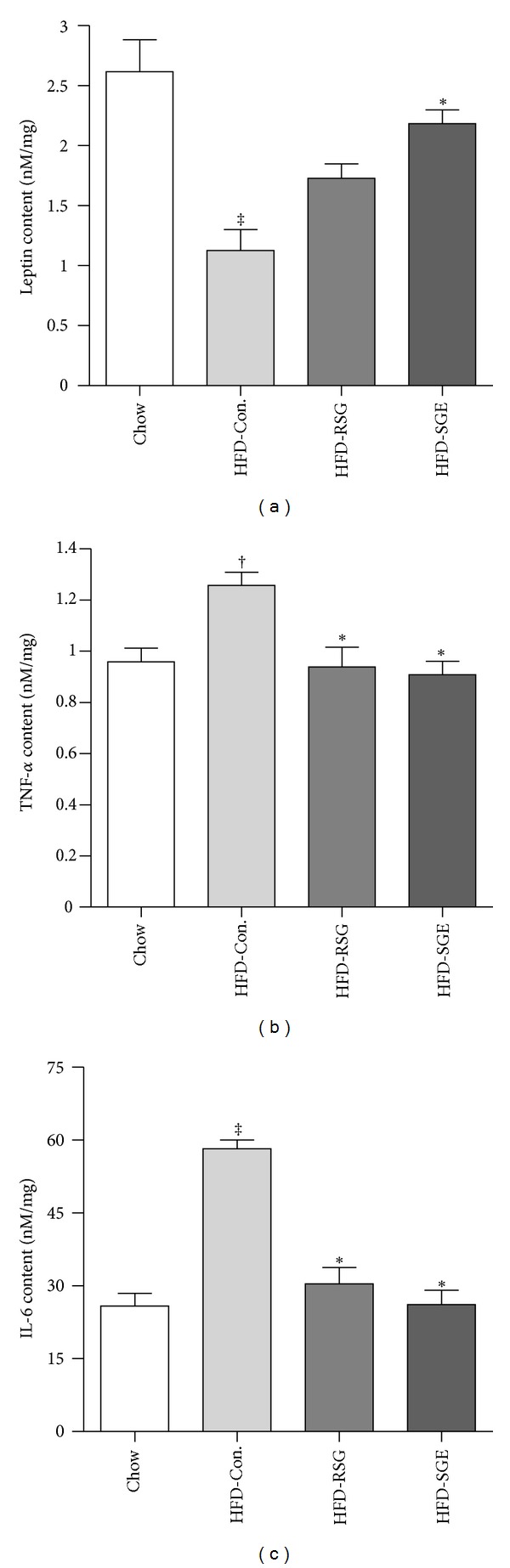
Effects of SME on leptin (a), TNF-*α* (b), and IL-6 (c) expression in adipose tissue of chow fed rats and HFD control rats treated with saline (5 mL/kg of body weight), and HFD rats treated with rosiglitazone (HFD-RSG, 3 mg/kg of body weight) or SGE (HFD-SGE, 2 g/kg of body weight). Adipose leptin, TNF-*α*, and IL-6 were determined using a multiplex rat adipocyte Linoplex kit stated in method section. Data are means ± SEM; *n* = 10 rats/group. ^†^ *P* < 0.05, ^‡^ *P* < 0.01 versus chow control; *P < 0.05 and **P < 0.01 compared with HFD-Con.

Leptin, TNF-*α*, and IL-6 were determined in adipose tissue of chow fed rats and HFD rats treated with saline, RGS, or SGE, respectively. As shown in Figure 2, HFD feeding significantly increased TNF-*α* by 26.5% and IL-6 by 49.8% and decreased leptin levels by 81.6% when compared with chow fed rats. Both SGE and RGS were capable of significantly inhibiting HFD-induced TNF-*α* and IL-6 (Figures 2(b) and 2(c), *P* < 0.05 and *P* < 0.01 versus HFD control, resp.). SGE treatment also significantly increased leptin expression (*P* < 0.05 versus HFD control) in adipose tissue of HFD rats.

### 3.4. SGE Improved Fatty Liver, Liver Steatosis, and Fibrosis

**Figure 3:**
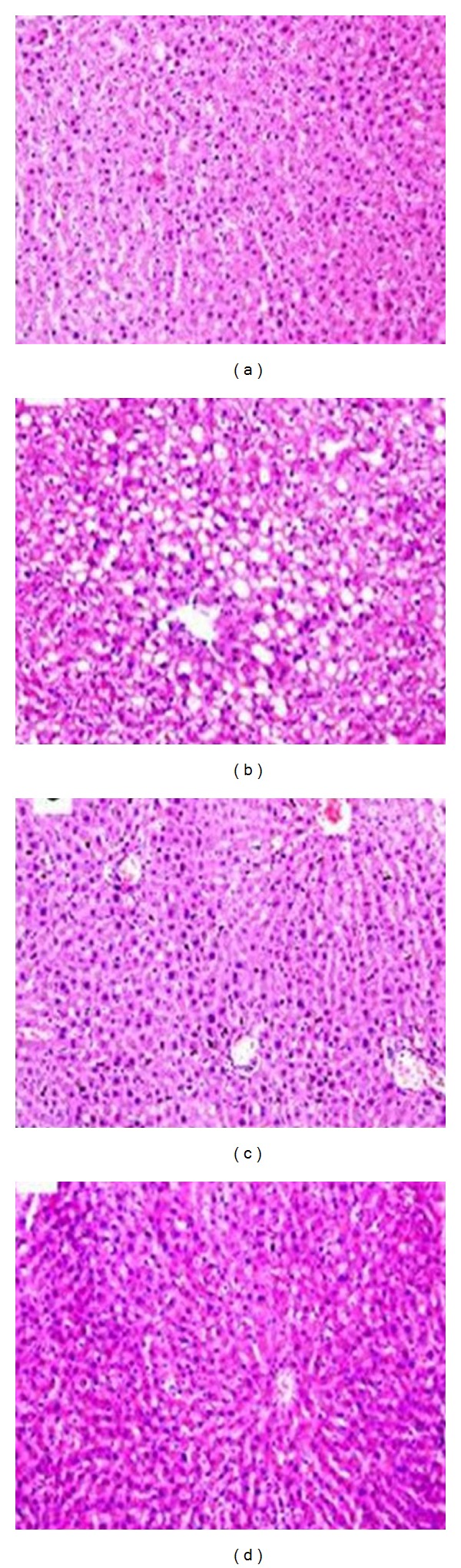
Representative images of hematoxylin and eosin (H&E) staining to visualize architecture of the liver and hepatocytes on sections of the liver section (6 *μ*m thick) from chow fed rats (a) and HFD control rats (b) treated with saline (5 mL/kg of body weight), and HFD rats treated with rosiglitazone (RSG, 3 mg/kg of body weight, (c)) or SGE (2 g/kg of body weight, (d)). All photomicrographs were taken at a magnification of 200x. The scale bar represents 50 *μ*M. Data are means ± SME, *n* = 6–8.

The photomicrographs of the H&E stain showed that HFD feeding increased hepatic fat deposits, evidenced by the majority of the hepatocytes of HFD rats that were distended by fat in comparison to the chow group (Figures 3(a) and 3(b)). The images of H&E stain also displayed macrovesicular steatosis, as many single large droplets had displaced the nucleus and ballooning degeneration causing conspicuous swelling of the cell and cytoplasmic vacuolation as shown in Figure 3(b). The treatment of HFD rats with RGS and SGE reduced fat liver depots (Figures 3(c) and 3(d)). The SGE groups showed histological features similar to the chow group with no macrovesicular steatosis as revealed in the HFD group as shown in Figure 3(b).

**Figure 4:**
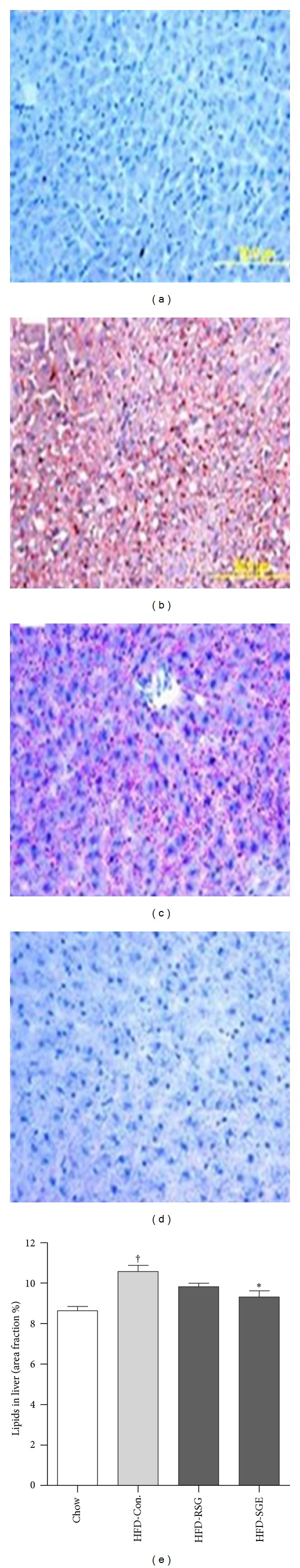
Representative images of Oil Red O (ORO) staining to visualize lipid droplets on liver sections (6 *μ*m thick) from chow fed rats (a) and HFD control rats (b) treated with saline (5 mL/kg of body weight), and HFD rats treated with rosiglitazone (RSG, 3 mg/kg of body weight, (c)) or SGE (2 g/kg of body weight, (d)). Images from the stained liver were taken at a magnification of 200x. The scale bar represents 50 *μ*M. Lipid droplets quantified at least 5 different high-power fields, being blind by two independent assessors. The intensity of staining of liver tissue with ORO provides a qualitative measure of the lipid contents (e). Data are means ± SEM, *n* = 6–8, ^†^ *P* < 0.05, ^‡^ *P* < 0.01 versus chow control; *P < 0.05 and **P < 0.01 compared with HFD-Con.

**Figure 5:**
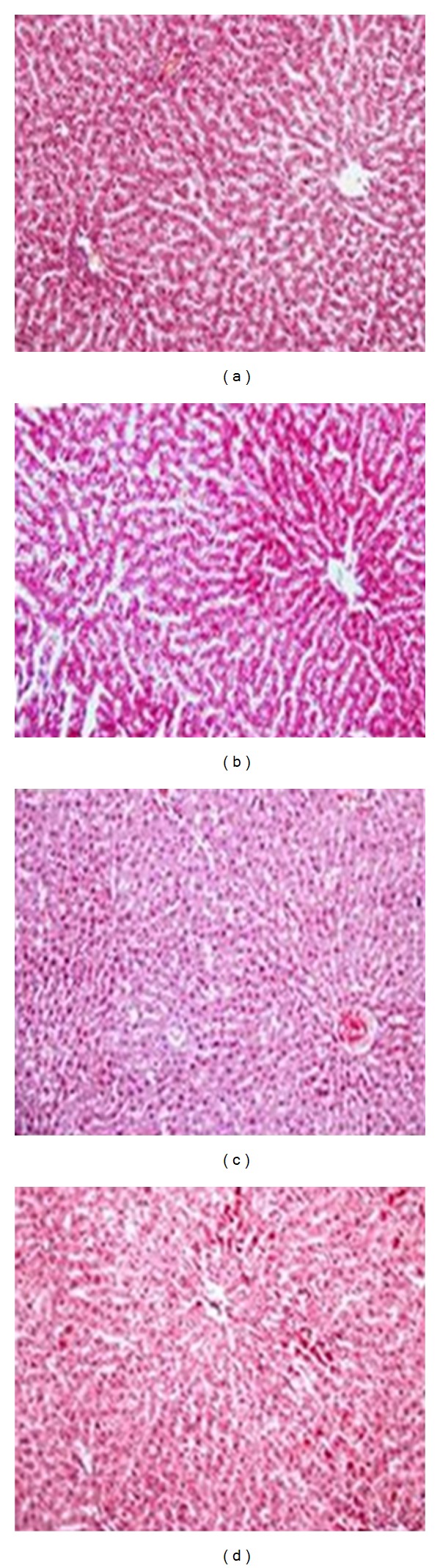
The photomicropictographs of Masson's Trichrome staining for assessment of the degree of fibrosis in the liver section from chow fed rats (a) and HFD control rats (b) treated with saline (5 mL/kg of body weight), and HFD rats treated with rosiglitazone (HFD-RSG, 3 mg/kg of body weight, (c)) or SGE (HFD-SGE, 2 g/kg of body weight, (d)). All photomicrographs were taken at 200x magnification.

ORO staining on frozen liver sections exhibited many lipid droplets in liver sections of HFD fed rats (Figure 4(b)), whereas a few lipid droplets were seen in the liver sections from the chow (Figure 4(a)) and SGE treated HFD rats (Figure 4(d)). Strikingly, there was an increase in the lipid content of liver tissue in HFD-RSG rats (Figure 5(c)). Analysis of blindly scored ORO-stained sections showed a statistically significant increase in the lipid content of liver tissue of HFD rats (Figure 4(e)). Levels of lipids were higher in the liver of HFD rats (10.77 ± 0.45%) compared with those in chow group (8.80 ± 0.34%). The RSG group had elevated levels of lipids (9.78 ± 0.21%), similar to the HFD fed group. The SGE treatment significantly lowered lipids values (9.10 ± 0.34%, *P* < 0.05 versus HFD control). 

In the photomicropictographs of Masson's Trichrome stain taken in the chow group displayed no fibrotic changes in the hepatocytes (Figure 5(a)). In the HFD group, intracytoplasmic fat was seen as clear vacuoles around the vessels and more distinct and slight fibrotic tissues were stained blue as shown in Figure 5B. In RSG treated HFD rats, fibrosis were observed around the vessels by the purplish green colour in comparison to chow fed rats (Figures 5(c) and 5(a)). SGE treated rats showed similar histological features in MT staining as chow fed rats (Figures 5(d) and 5(a)).

## 4. Discussion

As with our previous study, feeding a high-fat diet to SD rats led to visceral obesity associated with hyperlipidaemia, hyperinsulinaemia, and slight hyperglycaemia, which are characteristics of metabolic syndrome [11]. In the present study, we have demonstrated those ten weeks of high-fat diet feeding induced fatty liver disease in SD rats. The HFD fed rats assembled key biochemical features of MetS and NAFLD, including hyperinsulinaemia and increased HOMRE-IR value, marked elevation of hepatic enzymes, and hyperlipidaemia associated with increased TG accumulation in the liver. Histological evaluation remains the most important method of identifying NAFLD lesions, including steatosis, lobular and portal inflammation, hepatocyte injury as ballooning, and fibrosis [18]. In the present study, H&E, MT, and ORO stains on the liver samples revealed distended hepatocytes with increased lipid droplets, macro-vesicular steatosis, indicating the simple steatohepatitis and mild fibrosis with no cirrhotic displacements. The histological abnormalities in the HFD rats of this study were consistent with the findings of the previous literatures [19, 20]. High fat diet induced animal model of NAFLD has been widely used to identify the pathogenesis and evaluate new treatment for NAFLD [20, 21]. 

The elevated liver aminotransferase is a nonspecific clinical feature of NAFLD, which positively correlated to 90% patients with NASH [22]. Treatment with SGE for 4 weeks has proven to significantly normalise the liver hepatic aminotransferase (ALT and AST) to the level as normal chow control. Specifically, SGE treatment was effective in impeding fat infiltration (evidenced by decreased hepatic TG contents and lipid droplets) and preventing hepatic fibrosis, as shown in similar histological appearances as the chow control group. The results from biochemistry and histology assays demonstrated that SGE was protective against HFD-induced liver lesion and prevented NAFLD in HFD rats. 

NAELD's pathogenic mechanisms are still under investigation; however, fat accumulation, mainly triglycerides filtration within hepatocytes, is considered the first step in the development of NAFLD [23]. The clinical and animal studies demonstrated that levels of hepatic triglycerides are positively correlated to visceral obesity and insulin resistance [4, 24]. Under insulin resistant status, FFAs from lipolysis of visceral tissue are increased with decreased oxidative capacity. The elevated FFAs in the blood stream will directly circulate into the portal vein where the liver deposits FFAs as triglyceride in the hepatocytes and contribute to liver fibrosis. In addition to over-accumulation of triglycerides, *de no* synthesis of unoxidised fatty acids (ceramides and diglycerides) in the liver also increases, which trigger apoptosis of lipid-laden hepatocytes and impair insulin signal pathway. Therefore, aggressive treatment of hyperlipidaemia and inhibition of adipose lipolysis play a critical role in the overall management of patients with NAFLD and MetS. Statins are the first-line agents to treat hyperlipidaemia but there is a risk for liver injury in patients with NAFLD [25]. Ten weeks of HFD feeding in rats caused atherogenic dyslipidaemia with high levels of TG, low levels of HDL-C, and marked elevations in LDL-C concentrations. In this study, we showed that SGE had effects on hypertriglyceridemia and attenuated the elevated serum NEFAs, indicating that SGE may inhibit adipose lipolysis or enhanced FFAs availability. SGE also presented similar effects to RSG (insulin sensitizer) on hyperinsulinaemia and improved HOME-IR, but its potency was much less than RSG as the higher dose (2 g/kg of body weight) than RSG (3 mg/kg of body weight) was needed. SGE also raised HDL-C levels; however, the statistical significance was not achieved. Increasing the proportion of *S. miltiorrhiza* may be necessary if the formula would target at atherogenic dyslipidemia because the previous study has shown that *S. miltiorrhiza* markedly raised HDL-C in patients with ischemic cerebrovascular disease [12]. Specifically, we demonstrated that SGE had a beneficial effect on body weight and significantly reduced visceral fat mass but RSG treated rats gained more weight than HFD control. Thus, present study has proven that SGE had preventive effect on NAFLD as well as improved several metabolic abnormalities in HFD rats.

Accumulating data demonstrate that obesity and insulin resistance lead to NAFLD and hepatic fibrosis through not only fatty infiltration but also adipocytokines-induced inflammation [26]. Adipocytokines are cytokines secreted primarily by adipose tissue, including adiponectin, leptin, resistin, TNF-*α*, interleukins, and others. Decreased secretion of adiponectin and leptin from adipose tissue is involved in central obesity and insulin resistance [27]. Leptin is a key fat-derived hormone produced by adipose tissue, acting on the brain to increase satiety, hence inhibiting food intake and controlling weight gain [28]. After a high-fat meal, leptin secretion usually increased in rodents [29]. Our study showed that chronic HFD feeding was associated with reduced leptin expression in adipose tissue. This reduced leptin levels may contribute to the subsequent weight gain that we observed in the control HFD rats. We propose two mechanisms that may explain why a high dietary fat intake was associated with decreased leptin expression. Firstly, a long-term high-fat exposure (10 weeks) may overstimulate leptin secretion, hence depleting leptin contents in the adipose tissue. Secondly, increases in lipolysis have also been associated with decreased leptin synthesis in the previous study [30]. Decreased leptin synthesis may explain reduced leptin levels in adipose tissue after fat feeding because increased lipolysis has been documented after high-fat diets [31]. Our study has demonstrated that SGE treatment enhanced leptin expression, which is consistent with a recent study that circulating leptin was reversely associated with weight loss and VLDL-TG secretion in obese subjects [32]. Furthermore, the marked hepatic steatosis has been observed in leptin-deficient *ob/ob* mice [33]. Enhanced leptin expression may be one of mechanisms by which SGE ameliorated NAFLD and hepatic fibrosis in HFD rats. 

Overexpression of TNF-*α* and IL6 has been identified in the adipose tissue of obese patients [34]. TNF-*α* together with IL6 can mediate macrophage infiltration locally and at distant sites, such as liver. Hepatic inflammation resulting from adipose proinflammatory cytokines plays an important role in the development of NAFLD and progression of the fibrogenic process [35]. In the present study, HFD feeding significantly increased the weight of visceral fat and insulin resistance. In particular, decreased leptin and increased TNF-*α* and IL6 expression in visceral fat were found in HFD rats compared with standard chow fed rats. Treatment with both SGE and RGS significantly inhibited adipose TNF-*α* and IL6 expression but only SGE increased leptin expression in adipose tissue. Anti-inflammatory action may be one of the mechanisms that SGE attenuate HFD-induced liver pathology. 

The coexisting MetS and NAFLD provide a rationale for using a formula with multiple naturally occurring compounds that can target different pathological pathways involved in this complicated metabolic disorder, providing therapeutic effects via a spectrum of actions. SGE contains active compounds existing in *S. miltiorrhiza *such as 3, 4-dihydroxyphenyl lactic acid (named as Danshensu) and diterpenoid quinines (tanshinone IIA) from *S. miltiorrhiza,* and active compounds such as geniposide and genipin from *G. jasminoides*. The previous studies have demonstrated that the active compounds in *S. miltiorrhiz* ahave beneficial effects on the cardiovascular system through reducing oxidant stress, inhibiting inflammatory cytokine (plasminogen activator inhibitor-1), and improving lipid profiles [12, 13, 36]. The results in this study may also account for the pharmacological action of ingredients from *G. jasminoides*. Geniposide is one of the major iridoid glucosides in the fruit of *G. jasminoides*, which has been reported to possess anti-inflammatory activity [37]. A recent animal study has demonstrated that geniposide has an antiobesity effect, and the metabolite genipin shows a direct effect on the fatty liver through inducing expression of a lipid metabolism-related gene [15]. We have performed screening tests on many herbal extracts from single herb, but their actions were mild and lack of the capability to control most components of metabolic syndrome (data not published). The SGE used in this study have substantially exemplified that the effects of natural compounds together may provide synergistic effects. 

In summary, this study demonstrated that SGE improved circulating lipid profiles, reduced visceral fat, and improved insulin resistance, as well as effectively impeded the accumulation of triglycerides in the liver and fat infiltration in the hepatocytes with normal histological appearances and hepatic enzymes in HFD fed rats. SGE prevented the development of HFD-induced NAFLD maybe through attenuating release of inflammatory cytokine from adipose tissue. Because two herbs have been used in TCM for thousand years, it has been considered for its safety and tolerability. Large scale of clinical trials is worth being performed to prove that SGE is an optimal approach to the combination of non-alcoholic fatty liver disease and metabolic syndrome. 

## Figures and Tables

**Table 1:** Summary of metabolic parameters in chow and HFD fed rats with variety of treatments.

	Chow	HFD-Con.	HFD-RSG	HFD-SGE
Initial body weight, g	372 ± 16	414 ± 14^†^	409 ± 20	417 ± 16
Final body weight, g	412 ± 22	468 ± 19^†^	483 ± 32	438 ± 27*
Visceral fat mass, g	7.8 ± 1.2	14.60 ± 1.20^‡^	17.0 ± 1.4	8.9 ± 1.0**
Liver wt, g	11.7 ± 0.2	12.50 ± 0.90	13.3 ± 0.6	11.8 ± 0.6
Serum glucose, mM	6.75 ± 0.14	8.87 ± 0.24^‡^	7.65 ± 0.14*	8.15 ± 0.18
Serum insulin, mU	26.60 ± 0.22	46.25 ± 0.35^‡^	24.01 ± 0.18**	27.03 ± 0.31**
HOMA-IR	7.98 ± 0.09	18.23 ± 0.28^‡^	8.16 ± 0.17**	9.76 ± 0.29**
Serum TC, mM	3.98 ± 0.21	5.67 ± 0.24^†^	5.13 ± 0.32	4.35 ± 0.18*
Serum TG, mM	0.41 ± 0.03	1.03 ± 0.06^†^	0.59 ± 0.03*	0.51 ± 0.02**
Serum HDL-C, mM	1.20 ± 0. 22	0.86 ± 0.13^‡^	0.90 ± 0.16	0.96 ± 0.15
Serum LDL-C, mM	2.59 ± 0.52	4.34 ± 0.59^‡^	3.96 ± 0.67	3.16 ± 0.56*
Serum NEFA, mM	0.50 ± 0.10	0.77 ± 0.21^†^	0.57 ± 0.12*	0.58 ± 0.12*

Data are means ± SEM; *n* = 10 rats/group. ^†^ *P* < 0.05, ^‡^ *P* < 0.01 versus chow control; *P < 0.05 and **P < 0.01 compared with HFD-Con. HOMA is defined as (fasting insulin × fasting glucose)/22.5. Visceral fat included epididymal fat pad and inguinal fat. TC: total cholesterol; TG: triglycerides; HDL-C: high-density lipoprotein cholesterol; LDL-C: low-density lipoprotein cholesterol; NFFA: nonfree fatty acids.

## Abbreviations

NAFLD: Nonalcoholic fatty liver disease
HFD: High-fat diet
RSG: Rosiglitazone
SD: Sprague Dawley
SGE: *Salvia miltiorrhiza*, *Gardenia jasminoides* extracts.
